# Minocycline Improves the Efficacy of EGFR Inhibitor Therapy: A Hypothesis

**DOI:** 10.3389/fonc.2016.00231

**Published:** 2016-10-27

**Authors:** Ajit Venniyoor, Bassim Al Bahrani

**Affiliations:** ^1^National Oncology Center, The Royal Hospital, Muscat, Oman

**Keywords:** minocycline, EGFR inhibition, skin rash, PARP inhibitors, survival

## Abstract

Skin rash is a side effect of drugs that inhibit epithelial growth factor receptor (EGFR) as a part of targeted therapy of cancer. Its appearance and severity correlates with survival. Minocycline, an oral tetracycline antibiotic, is recommended as treatment (and increasingly, for prevention) of the rash, though infection is seen in only one-third of the patients. Minocycline has additional anticancer properties such as poly(ADP-ribose) polymerase inhibition. It is proposed that such properties contribute to the efficacy of EGFR inhibitors and can also explain the positive correlation between grade of rash and survival as patients with higher grades of rash are more likely to receive minocycline. Early concurrent administration of minocycline is recommended in patients planned for EGFR therapy while awaiting trials proving this hypothesis.

Inhibition of epithelial growth factor receptor (EGFR) with either monoclonal antibodies (cetuximab, panitumumab) or tyrosine kinase inhibitors (erlotinib, gefitinib) is approved for the treatment of some subsets of cancers of colon, lung, pancreas, and head and neck. One of the major side effects of these drugs is an acne-form skin rash (1). Multiple trials have shown that the appearance and the degree of rash are associated with improved survival (2, 3). It is recommended that higher degrees of rash be treated with tetracyclines, such as minocycline (1), despite studies suggesting that only about 40% of them have an infection (4).

Minocycline, in addition to being an antibiotic, has multiple other actions (5). It is a poly(ADP-ribose) polymerase-1 (PARP-1) inhibitor (6), and there is evidence that inhibition of PARP pathway could be useful in EGFR-dependent tumors. EGFR mutants have lower levels of BRCA1 expression (7) and have deficiencies in the homologous recombination repair pathways that make them sensitive to another PARP inhibitor, such as olaparib (8). In triple-negative breast cancer cell lines, a combination of EGFR and PARP inhibition have been shown to increase synthetic lethality (9). Synergy of EGFR and PARP inhibition has been reported on cell lines (10, 11), and data on combination of PARP inhibitors with anti-EGFR monoclonal antibodies has been reviewed (12). A trial is currently recruiting head and neck cancer patients in a Phase I trial for the combination of cetuximab and olaparib (NCT01758731) (13).

Interestingly, minocycline was chosen for the treatment of skin rash in the first randomized trial (conducted by Memorial Sloan Kettering Cancer Centre) for its anti-inflammatory rather than anti-infective effects (14).

Minocycline also appears to enhance the antitumor effects of 5-FU in tumor CT-26 xenograft mice (15). Therefore, it is possible that the increased activity of EGFR inhibitors and correlation with degree of skin rash can be attributed partly to concurrent use of minocycline.

Interestingly, the recently published Pan Canadian Rash trial (16) on the use of minocycline in the management of skin rash caused by erlotinib in lung cancer showed a trend toward improved survival associated with the early use of minocycline (7.6 and 8 months in prophylactic and reactive use) versus if used only when severe rash appears (6 months). Thus, this hypothesis is worth exploring in prospective randomized trials. Analysis of existing data generated in Phase III trials using EGFR inhibitors with specific focus on survival versus minocycline use could also provide evidence supporting this hypothesis.

In the meantime, the use of minocycline should be recommended in patients on EGFR inhibitors even before the appearance of skin rash [in line with current recommendations (17)], for a possible survival effect. The antibiotic has been used over prolonged periods (3–4 months) for the treatment of acne vulgaris, with hyperpigmentation and a lupus-like syndrome as side effects (18). Repurposing of (benign) drugs for their anticancer effects is an active field of research, and minocycline appears to be an active drug for inclusion in this list (19).

## Author Contributions

Drs. AV and BB: conceived, wrote, and approved.

## Conflict of Interest Statement

The authors declare that the research was conducted in the absence of any commercial or financial relationships that could be construed as a potential conflict of interest.
